# Brief and Telehealth Acceptance and Commitment Therapy (ACT) Interventions for Stress in Inflammatory Bowel Disease (IBD): A Series of Single Case Experimental Design (SCED) Studies

**DOI:** 10.3390/jcm11102757

**Published:** 2022-05-13

**Authors:** Joseph Lavelle, Darragh Storan, Varsha Eswara Murthy, Noemi De Dominicis, Hugh E. Mulcahy, Louise McHugh

**Affiliations:** 1 School of Psychology, University College Dublin, D04 V1W8 Dublin, Ireland; varsha.eswara-murthy@ucdconnect.ie (V.E.M.); louise.mchugh@ucd.ie (L.M.); 2 Department of Gastroenterology, Saint Vincent’s University Hospital, D04 T6F4 Dublin, Ireland; dstoran@hotmail.com (D.S.); noemidedominicis@svhg.ie (N.D.D.); hugh.mulcahy@ucd.ie (H.E.M.); 3 School of Medicine, University College Dublin, D04 V1W8 Dublin, Ireland

**Keywords:** inflammatory bowel disease (IBD), acceptance and commitment therapy (ACT), brief interventions

## Abstract

Psychological intervention targeting distress is now considered an integral component of inflammatory bowel disease (IBD) management. However, significant barriers to access exist which necessitate the development of effective, economic, and accessible brief and remote interventions. Acceptance and commitment therapy (ACT) is a therapy with demonstrated acceptability and a growing evidence base for the treatment of distress in IBD populations. The present paper trialled two brief ACT interventions via randomized multiple baseline designs. Study 1 trialled a single-session ACT intervention (delivered face-to-face and lasting approximately two hours) targeting stress and experiential avoidance, respectively. Participants were seven people with an IBD diagnosis who presented with moderate to extremely severe stress (five females, two males; *M* age = 39.57, *SD* = 5.74). The findings of study 1 indicate that a single-session ACT intervention represented an insufficient dosage to reduce stress and experiential avoidance. Study 2 investigated a brief telehealth ACT intervention (delivered via a video conferencing platform and lasting approximately four hours) targeting stress and increased psychological flexibility. Participants (*N* = 12 people with an IBD diagnosis and mild to extremely severe stress) completed baselines lasting from 21 to 66 days before receiving a two-session ACT telehealth intervention supplemented by a workbook and phone consultation. Approximately half of participants experienced reduced stress, increased engagement in valued action, and increased functioning. Despite shortcomings such as missing data and the context of COVID-19, the present findings suggest that brief ACT interventions in this population may be effective and economic, though further research and replications are necessary.

## 1. Introduction

Inflammatory bowel disease (IBD) is a diagnosis encompassing numerous chronic diseases impacting the human bowel, including Crohn’s disease (CD) and ulcerative colitis (UC). The impact of IBD is wide-ranging and symptoms include diarrhoea, rectal bleeding, unintended weight loss, and fatigue, while IBD also confers an increased risk of certain cancers [1,2]. Co-morbid psychological difficulties are also prevalent, including elevated stress, depression, anxiety, and body image impairment [3,4,5,6,7]. This further exacerbates the impact on the individual’s quality of life, but also impacts disease management. Indeed, comorbid psychological difficulties are associated with disease activity, flares, treatment response, and the necessity of surgical intervention [2,6,8,9,10,11,12,13,14,15]. Growing evidence suggests that stress—in particular—is associated with disease activity and severity while conflicting evidence suggests that stress may also be involved in the initial development of IBD [9,15]. Given such convincing evidence of a psychological component and impact in and of IBD, psychological support is now considered an integral component of modern IBD management [16,17].

Myriad interventions are available to intervene with stress in those diagnosed with IBD, ranging from psychoeducational programs, stress management and relaxation strategies, hypnotherapy, mindfulness-based stress reduction treatments, and cognitive behavioural therapy (CBT) [18,19]. Gracie et al. and Knowles et al. suggested a varying rate of effectiveness of these interventions, with psychoeducation and CBT tending to convey the greatest improvement in psychological distress and wellbeing. Moreover, the benefits of psychological interventions tended not to extend to disease-related outcomes (e.g., disease activity and severity). However, the findings of Gracie et al. and Knowles et al. contrast somewhat with those of earlier reviews, which observed minimal-to-no effectiveness of psychotherapeutic interventions in intervening with depression, quality of life (QoL), coping, and anxiety [20,21]. A concern common to reviews to date centres on methodological rigor and reporting. Many studies are observed to be non-randomized, devoid of inclusion and exclusion criteria, neglect to report salient participant data (e.g., IBD type), and neglect to report sufficient intervention program detail [18,19,20,21].

More recently, studies have investigated the impact of acceptance and commitment therapy on psychological suffering in those experiencing chronic health conditions. Acceptance and commitment therapy (ACT) is a process-orientated therapy that proposes psychopathology or psychological suffering emerges from psychological inflexibility whereby individuals’ efforts to control, escape, and avoid unwanted thoughts and feelings act as a barrier to valued and effective behaviour [22]. ACT aims not to change or reduce distressing and unwanted internal experiences, but to shape ways of limiting the influence of those thoughts and feelings on day-to-day living and goal achievement by increasing psychological flexibility [23]. Therefore, ACT targets ineffective and unhelpful behaviour which functions to avoid internal events (e.g., unwanted thoughts, emotions, and stress), and introduces mindfulness and acceptance-based skills to facilitate behaviour change and reduce psychological suffering [22,24]. Growing evidence has supported the effectiveness of ACT for myriad psychological and health conditions including anxiety, depression, stress, chronic pain, irritable bowel syndrome (IBS) and smoking cessation [25,26,27,28,29,30].

Initial evidence suggests ACT may be an appropriate intervention to target psychological suffering in people with an IBD diagnosis. For example, the ACT process of cognitive fusion—the tendency to perceive and act on thoughts through a context of literality—has been observed to be negatively associated with psychological and physical health in IBD and mediates the relationship between chronic-illness-related stigma and psychological QoL [31,32]. Moreover, changes in cognitive fusion rather than IBD symptomatology predicted improvements in psychological health. Similar findings have been observed for experiential avoidance, with experiential avoidance mediating the relationship between IBD symptomatology and physical and psychological QoL [33]. ACT interventions, particularly brief ACT interventions, appear to be acceptable to people with IBD and other chronic health conditions [34,35,36]. Lastly, the first trials of ACT in people with IBD suggest it may be effective in intervening with psychological distress [34,37]. The latter, Wynne et al., investigated the effectiveness of an eight-week ACT group targeting psychological distress in people with IBD. They observed that the ACT intervention produced reductions in stress and depression while also resulting in increased QoL and psychological flexibility. Indeed, early evidence suggests ACT is an appropriate, effective, and acceptable model to intervene with distress in IBD.

Despite the promise of interventions such as those employed by Wynne et al., such intensive and medium- to long-term interventions may not always be suitable. Reasons for this range from limited clinical and economic resources in healthcare systems, willingness to access psychological support, and feasibility for people with IBD themselves [38]. Such challenges have influenced the development of accessible, brief, low-intensity, and self-directed interventions for a range of psychological concerns. Indeed, growing evidence suggests briefer interventions can effect substantial change for clients. For example, almost half of clients with depression and anxiety exhibit sudden large gains within the first two to four CBT treatment sessions [39,40]. Similar gains are observed in CBT for post-traumatic stress disorder (PTSD; 53 percent of clients), adolescent depression, binge eating (62 percent of clients), and irritable bowel syndrome (30 percent of clients) [41,42,43,44]. Furthermore, this rapid response is associated with long-term improvements in functioning and a reduction in long-term relapse rates [39,43,45]. Similar gains have been observed in brief ACT interventions. For example, 90 min and three-hour focused acceptance and commitment therapy (FACT) interventions produced substantial and lasting reductions in depression which were comparable to a one-day (i.e., six-hour) FACT workshop [46,47]. Similar effects have been observed following brief ACT interventions for other presented concerns, including repetitive negative thinking and psychotic depression [48,49]. While little attention has been devoted to brief ACT interventions for IBD, early evidence suggests they are acceptable and potentially effective [34,35,36,39]. Taken as a whole, these findings suggest that for many individuals, significant treatment benefits are conferred after as little as two to four hours of intervention, while such brief interventions (including brief ACT interventions) appear acceptable to people with long-term health conditions including IBD.

Given the barriers to extended forms of psychological support, brief and low-intensity interventions are needed which are relatively low-cost, evidence-based, and produce reductions in stress, depression, and anxiety in people with IBD that are clinically meaningful. The present paper intended to understand the required dosage to reduce psychological distress in people with comorbid IBD. Given its significant prevalence and impact on QoL and disease activity, stress was given the greatest focus with a lesser focus on depression and anxiety. Under the present research, two cohorts of patients with an IBD diagnosis were delivered a single-session and two-session ACT intervention, respectively. Both interventions were trialled via single-case experimental designs (SCEDs) which facilitate the investigation of individual benefit and risk conferred by the brief ACT interventions and generalizable intervention effectiveness. Given existing findings on ACT for IBD populations and brief ACT interventions, it was predicted that the interventions would produce reduced stress and increases in psychological flexibility on daily ecological-sampling-style measures of both and validated questionnaires measuring stress [35,37,46,50]. It was predicted that the interventions would also lead to reductions in depression.

## 2. Materials and Methods

The following describes two studies trialling a single-session and two-session telehealth ACT intervention, respectively, and commences with methodological and analytic detail shared by both studies. This is followed by methodological detail, results, and discussion specific to studies one and two, respectively.

### 2.1. Design

The present study employed a single-case experimental design (SCED) wherein each participant is treated as an individual study. In doing this, SCEDs provide evidence of the effectiveness of interventions, drawing on substantially fewer participants than nomothetic approaches (such as RCTs) [51,52,53,54,55,56,57,58]. The specific SCED employed was randomized multiple baseline designs with the temporal staggering and randomized onset of intervention. Under this design, experimental control is demonstrated when change is evident in the data across participants with staggered intervention starting points [59]. The independent variable for each participant was the pre–post intervention stage. As per Kratochwill and Levin, the onset of the ACT intervention was randomized across a 5- to 7-day period [60]. The dependent variables were stress and psychological flexibility (experiential avoidance in study 1). Participants reported stress and psychological flexibility daily for a minimum of two weeks and three weeks, respectively, before intervention and for up to 10 weeks post-intervention via two to four Likert-style items.

### 2.2. Randomization

The temporal onset of ACT interventions was randomized with the aid of the web-based tool “Research Randomizer” [61]. Randomization was performed by the first author while naive to the participant’s stress scores or clinical presentation. On occasion, it was necessary to breach the randomized order of intervention delivery to ensure the timely progression of the research and to minimize the burden on participants. Typically, this occurred due to participant illness and/or hospitalization.

### 2.3. Institutional Context and Ethics

The present SCEDs were conducted at a single academic teaching hospital in Dublin, Ireland, between October 2019 and August 2021. The teaching hospital in question hosts the Centre for Colorectal Disease and is considered a centre of excellence in the treatment and management of colorectal diseases including IBD. At the time of participation, all participants received care at the hospital’s gastroenterology service.

Ethical approval was granted by the institutional ethics committee (at Saint Vincent’s University Hospital Dublin, Dublin, Ireland) and exemptions from full ethical review were granted at University College Dublin, Dublin, Ireland (HS-E-19-112; HS-E-21-63).

### 2.4. Inclusion and Exclusion Criteria

Prospective participants aged 18–65 with mild stress or above as measured by the DASS-21 and a histological and radiological diagnosis of Crohn’s disease or ulcerative colitis were eligible to participate [62]. Prospective participants were excluded from participation if they had previously participated in research trialling an ACT intervention.

### 2.5. Recruitment

Recruitment was led by IBD specialists at the above-named academic teaching hospital in September 2019 and January 2021. Said IBD specialists were briefed by the first, second, and second-last authors regarding inclusion and exclusion criteria and participation activities. Said IBD specialists subsequently broached participation with those attending their clinics. It was made clear to all prospective participants that refusal to participate would not impact their care in any manner. Those who remained interested were screened per the stress inclusion criteria with those eligible being permitted a further week to consider participation.

### 2.6. Measures of Stress

Two measures of stress were employed in both experiments, namely DASS-21 and an adapted “Stressometer”. DASS-21 was administered on three occasions: screening, week zero (immediately before study commencement; pre-study from hereon in), and at the end of the study. The adapted “Stressometer” was completed daily in an ecological-sampling-style format with the measure and response delivered via SMS [50].

#### 2.6.1. Depression Anxiety Stress Scale (DASS-21)

The DASS-21 was employed as a measure of stress, depression, and anxiety. The measure includes three seven-item scales assessing each construct. Respondents report the extent to which they experienced various symptoms of stress, depression, and anxiety over the past week on a four-point Likert scale. Responses ranged from 0 (did not apply to me at all) to 3 (applied to me very much or most of the time) with the measure taking approximately ten minutes to complete. The measure has been observed to be valid, reliable (depression subscale α = 0.96, anxiety subscale α = 0.89, and stress subscale α = 0.93), and have a well-supported three-factor structure, and has been trialled in clinical and non-clinical populations [62,63,64,65].

#### 2.6.2. Adapted Stressometer

An adapted form of the “Stressometer” was employed to assess stress daily in an ecological-sampling-style manner. As such, the measure was intended to capture stress in vivo while also minimizing the assessment burden (taking less than one minute to complete). The “Stressometer” is a validated and responsive measure of stress in people with IBD [66]. The adapted measure read as follows:

*Please reply with the number between 0 and 10 that best describes the stress you have experienced today (where a score of 0 indicates “No stress” and a score of 10 indicates “Extreme Stress”).*

### 2.7. Treatment Integrity

Intervention protocols were developed by the first, third, and last authors. All three have experience in delivering ACT interventions, and authors one and three have completed 18-month trainings in ACT and mindfulness-based interventions (MBIs) and training in counselling and clinical skills. The last author is a peer-reviewed ACT trainer who provided their expertise during protocol development and provided supervision to the first author during study 1 and to the first and third authors during study 2.

In addition to the above steps to maximize treatment integrity, audio recordings of intervention sessions were kept with participant consent. Randomly selected sessions were subsequently assessed for treatment fidelity using the ACT Fidelity Measure (ACT-FM) [67]. The assessor (a member of the Contextual Behavioural Science lab coordinated by the last author but not an author of the present manuscript) was also experienced in ACT and had completed an 18-month training in ACT and MBIs. Sessions were rated as demonstrating a high presence of ACT-consistent interventionist behaviour and a low presence of ACT-inconsistent behaviour.

### 2.8. Analytic Strategy

#### 2.8.1. SCED Analysis

SCED analyses were conducted using the R packages SCED and metafor, which facilitates accessible and robust visualization, analysis, and meta-analysis of A-B SCED data while minimizing researcher degrees of freedom [68,69]. A full discussion of the analyses carried out via the R SCED package is available via Hussey and Eswara Murthy et al., and will be summarized here [68,70]. To enable visual assessment of between-phase difference, baseline trends, and phase variability, raw data including OLS regression trend lines, median values per phase, and median absolute deviation of scores within each phase were plotted through an in-built function in the SCED package. Given the poor inter-rater reliability of visual assessment of intervention effects through visual analysis, the SCED package also permits quantitative analysis of data [71,72]. As such the package calculates *p* values via robust, non-parametric permutation tests and calculates three robust effect sizes (for a primer on commonly reported effect sizes in SCED research see Parker et al.): median difference between conditions, Ruscio’s A (also known as the Common Language Effect Size, the Probability of Superiority, and Nonoverlap All Pairs), and Hedge’s *g* [73,74,75,76,77,78,79].

Finally, a random-effects meta-analysis was conducted via the R packages SCED and metafor [68,69]. Following standard meta-analytic practices, the SCED package calculates 95% confidence intervals on the meta effect size, metrics of heterogeneity, and 95% credibility intervals on the effect size [68,70].

#### 2.8.2. Clinically Significant Change (CSC)

Clinically significant and reliable changes in depression, anxiety, stress, experiential avoidance, and psychological flexibility were assessed per Jacobson and Truax’s (1991) guidelines via The Leeds Reliable Change Indicator [80,81,82]. Reliable and clinically significant changes were calculated using available psychometric and normative data. Reliable change was observed wherein a participant’s score was increased or reduced by 1.96 standard deviations at post-study. CSC was achieved when a reliable change was observed, and the participant’s score had crossed a cut-off point denoting that their post-intervention score on the measure in question was now closer to the mean of a non-clinical population than that of a clinical population.

#### 2.8.3. Missing Data

Missing data varied across studies and participants from 0% missingness to 35% missingness. Data analysis was conducted using available data. Given the substantial number of datapoints (59 and 101 per participant in studies 1 and 2, respectively) it was deemed that there were sufficient data to assess the effect of the intervention despite the presence of missingness. Moreover, a clear consensus to address missing data in SCEDs does not yet exist nor does it feature under the SCRIBE reporting guidelines or risk of bias tools for SCEDs [83,84,85,86].

## 3. Study 1: Randomized Multiple-Baseline Design Trial of a Single-Session ACT Intervention

### 3.1. Study 1 Materials and Methods

#### 3.1.1. Participants

**Figure A1:**
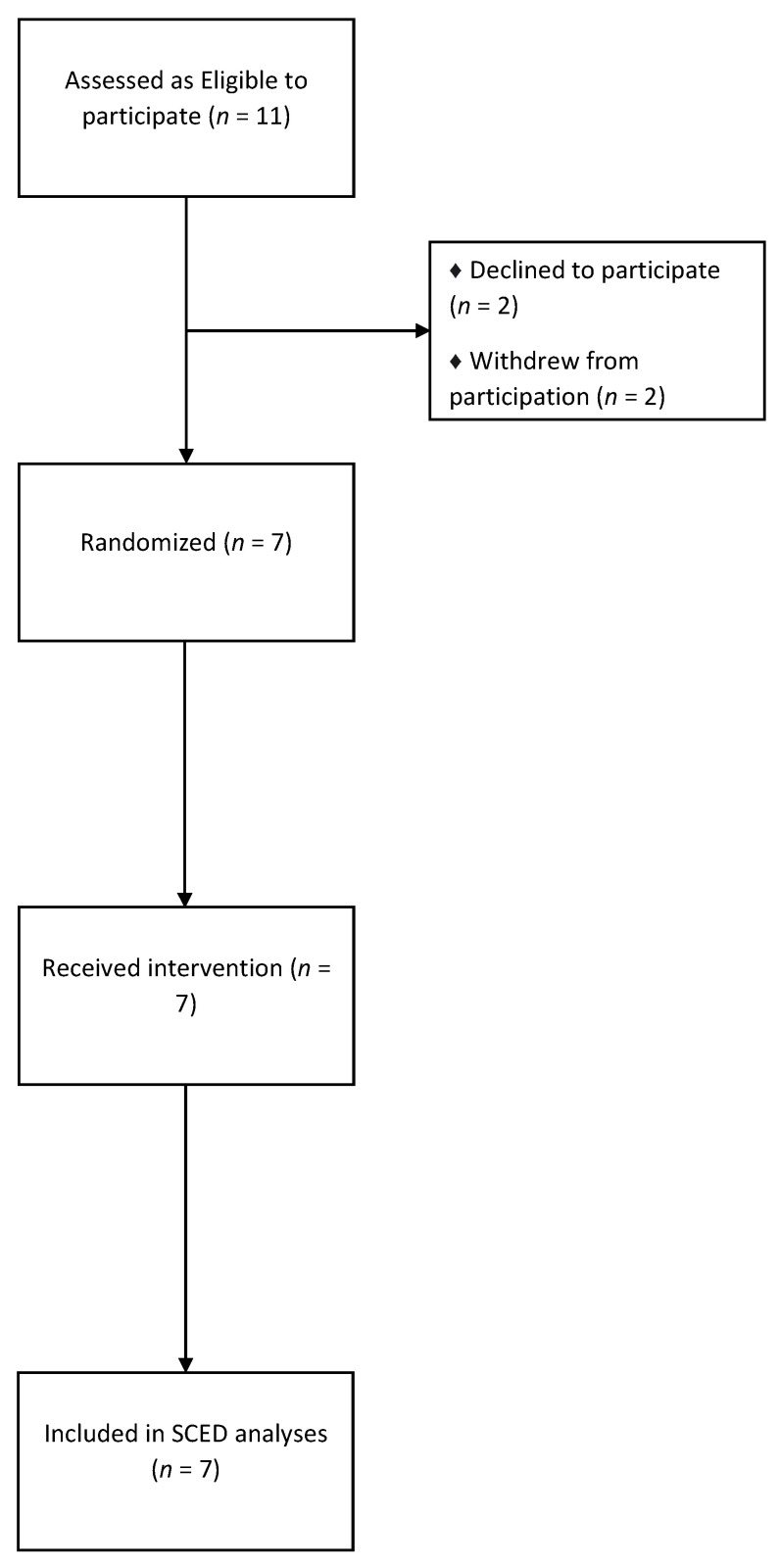
Study 1 participant flowchart.

The recruitment strategy described under Section 2.5 identified eleven prospective participants (7 females, 4 males; *M* age = 36.18 years, *SD* = 7.99) who were invited to participate. Of these eleven participants, two chose not to participate after consulting the information pack. Two further participants provided informed consent but dropped out during the baseline assessment phase (i.e., before receiving the intervention) citing work and education commitments as reasons for doing so. The final sample consisted of seven participants scoring in the moderate to the extreme range for stress on the DASS-21 (5 females, 2 males; *M* age = 39.57, *SD* = 5.74). Further participant details and baseline characteristics are available in Table 1, and recruitment is summarized in Figure A1 (Appendix A).

#### 3.1.2. Measures of Experiential Avoidance

In addition to the measures of stress employed under Section 2.6, two measures of experiential avoidance were employed. These were the Acceptance and Action Questionnaire-II (AAQ-II; delivered week zero and at the end of the study) and a single-item measure of EA designed for daily administration with a low assessment burden [70,87].

##### Acceptance and Action Questionnaire-II (AAQ-II)

The AAQ-II is a widely used seven-item measure of PF (taking approximately five minutes to complete) which asks respondents to rate various statements pertaining to EA on a seven-point Likert scale. Items include various statements reflecting inflexibility and experiential avoidance, such as “I’m afraid of my feelings”, and rated on the extent to which they are true (1 = Never True; 7 = Always True) [87]. The AAQ-II has been used extensively across research and practice, adapted for different languages and cultures, and used with diverse population groups including people with IBD [37,88]. While initially considered reliable (internal consistency; α = 0.88) and valid, recent queries have been raised about its discriminant validity [89,90].

##### Daily Experiential Avoidance (EA) Measure

An item to measure experiential avoidance (EA) was drawn from the third author’s research on a single-session ACT intervention for people experiencing homelessness [70]. The measure was designed to capture EA while minimizing the burden on participants (taking approximately one minute to complete) due to daily assessment across 59 days and maximizing consistent responding. As per the AAQ-II, higher scores denote greater EA. The measure read as follows:

*On a scale of 1–10, where 1 indicates “not at all” and 10 being “very much so”, please respond to the question below: Have your thoughts and feelings blocked you”.*

#### 3.1.3. Acceptance and Commitment Therapy (ACT) Intervention Protocol

The ACT intervention protocol was developed by the first and last authors and was informed by previous research employing ACT for IBD and single-session ACT interventions [9,34,35,37,70]. While all six core processes of ACT were targeted and taught, particular emphasis was placed on undercutting cognitive fusion and experiential avoidance given their contribution to suffering and distress in people with IBD [31,33]. The full intervention protocol is available via the first author’s OSF page (see Supplementary Materials) and is summarized in Table 2.

The intervention was delivered face-to-face via one session lasting approximately two hours in duration. The initial phase of the intervention session described what the participant could expect of the two-hour session, introduced the therapist, and endeavoured to establish rapport. This was followed by psychoeducation on stress and its role in IBD. This segued into a discussion of ineffective behaviour centred on control and avoidance of unwanted internal events such as thoughts, feelings, and physical sensations. This was supplemented with a worksheet that asked the participant to identify their attempts to control internal experiences and the consequences of the same.

Phases three and four of the intervention introduced participants to the notion of accepting their unwanted experiences including thoughts, emotions, and physical sensations. Phase five entailed values clarification via a values card sort to establish values as motivators of consistent and long-term behaviour change. Phase six endeavoured to summarize and integrate the previous intervention components. The “ACT Matrix” was used to identify unwanted internal experiences surrounding a chosen life domain, recognize control-oriented strategies the participant may be engaging in, and finally identify a series of short- and long-term goals to operationalize the values identified in phase 5 [91]. Finally, phase 7 introduced a guided perspective-taking exercise known as “the compassionate hand” which aimed to connect the participant with a sense of self-compassion. In addition to the above, participants were provided with Supplementary Materials to facilitate the implementation of the skills and processes shaped in the session.

#### 3.1.4. Procedure

Participants were recruited via a single academic teaching hospital in Dublin, Ireland per the recruitment strategy and inclusion criteria (Section 2.4 and Section 2.5 respectively). Participants who provided informed consent completed the DASS-21 and AAQ-II on week 0 and post-study. From days 1–59 participants provided daily reports of stress and EA via the single-item measures described under Section 2.6.2 and Section “Daily Experiential Avoidance (EA) Measure”, respectively. Single-item measures of stress and EA were delivered via a single SMS message at 6.00 p.m. GMT daily and sent using a research phone operated by the first author. Participants were encouraged to provide labelled responses via single SMS-message replies.

From day 15 onwards, participants began to receive the single-session ACT intervention per the preset randomized order. Intervention sessions were scheduled at a time amenable to the participant by the second author. Intervention sessions were delivered by the first author per the protocol (Section 3.1.3) using facilities at the host hospital.

### 3.2. Study 1 Results

#### 3.2.1. Missing Data

Minimal missing data were observed in daily measures of stress and PF. Participants 4, 5, and 10 missed one data session each (i.e., did not report stress or PF) in phases A and B respectively. Two participants (participants 4 and 5) did not complete the DASS-21 and AAQ-II post-study.

#### 3.2.2. Stress and EA as Measured by Daily Single Items

**Figure 1:**
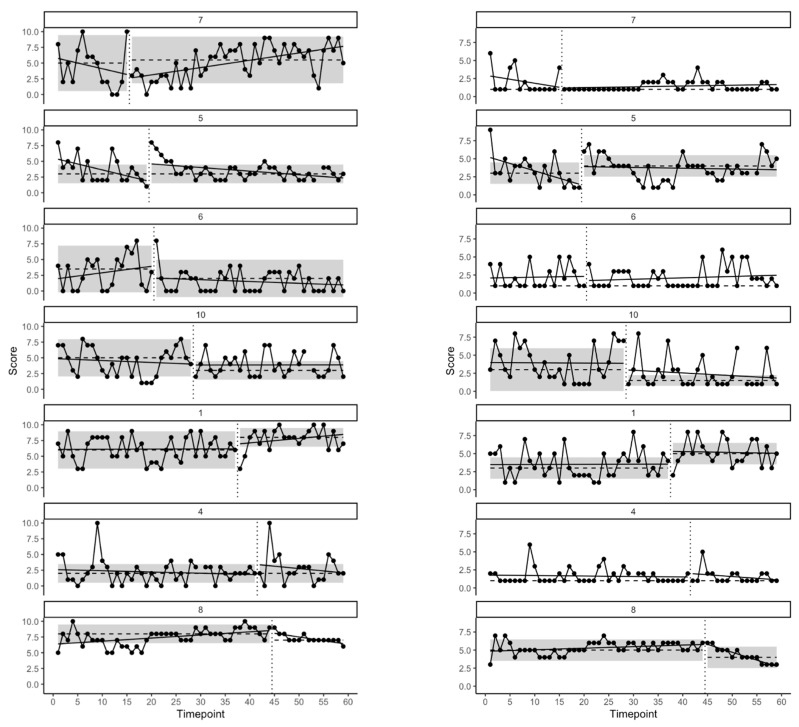
SCED data for all participants on daily single measures of stress (**left**) and EA (**right**). Participant data are ordered via the order in which they received the intervention. Vertical dotted lines denote the data point at which the intervention was delivered. Shading denotes the one median absolute deviation from the median. Dashed horizontal lines denote the median while solid lines denote within-phase OLS regression trend lines.

Data for both the stress and EA variables are presented in Figure 1. Participant data are ordered per the order in which they received the ACT intervention to visualize the impact of the intervention across multiple baselines. Following visual inspection of the plots there appeared to be little discernible change in stress attributable to the ACT intervention, with one participant appearing to experience reduced stress (participant 6) while one appeared to experience increased stress (participant 1). Meanwhile, two participants appeared to experience reduced EA (participants 8 and 10) while one appeared to experience increased EA (participant 1).

Following quantitative analysis of stress data, it was observed that one of seven participants (see Table 3; participant 6; 14%) demonstrated reduced stress following the ACT intervention that was statistically significant. Furthermore, one of seven participants (participant 1; 14%) demonstrated increased stress that was statistically significant. These data suggest minimal impact on stress as a function of the intervention with the conclusion strengthened further via meta-analysis across participants, *P*(A < B) = 0.511, 95% CI [0.409, 0.613], 95% CR [0.290, 0.729], *p* = 0.8287, generalized OR = 1.05, 95% CI [0.69, 1.58], 95% CR [0.41, 1.58].

Quantitative analysis of EA data demonstrated that three of seven participants saw reduced EA (see Table 3; participants 7, 8 and 10; 42%) while one of seven participants experienced increased EA (participant 1; 14%). In the case of participant 7 this may be an artefact emerging from the minimal variability in their EA data and long B phase rather than a function of the intervention. Meta-analysis across participants suggested little generalizable effect on EA because of the intervention, *P*(A < B) = 0.471, 95% CI [0.342, 0.603], 95% CR [0.185, 0.777], *p* = 0.6665, generalized OR = 0.89, 95% CI [0.52, 1.52], 95% CR [0.23, 1.52].

#### 3.2.3. DASS-21 Measured Depression, Anxiety, and Stress

DASS-21 scores for participants at pre and post study are presented in Table 4. Three participants (participants 7, 8 and 10) demonstrated reliable improvements in DASS-21 measured stress and one (participant 7) experienced clinically significant reductions in stress. Participant 8 demonstrated reliable and clinically significant improvements in depression. Two participants (participants 7 and 8) demonstrated reductions in anxiety that denoted both reliable and clinically significant improvement. Zero participants demonstrated deteriorations in stress, depression, or anxiety that were reliable or clinically significant.

#### 3.2.4. AAQ-II Measured Experiential Avoidance

Five participants reported AAQ-II measured EA at both time points with no participants demonstrating reliable or clinically significant change. Their scores are summarized in Table 5.

### 3.3. Study 1 Brief Discussion

Study 1 aimed to investigate the effectiveness of a brief one-session ACT intervention to reduce stress and experiential avoidance in a sample of individuals with an IBD. Given the design, it was possible to detect both improvements and deterioration following exposure to the single-session intervention. Regarding stress, one participant (participant 6) demonstrated reduced stress as measured by the adapted “Stressometer”, and three participants demonstrated reduced stress on DASS-21 (participants 7, 8, and 10). Of note, the same three participants demonstrated reduced EA on the daily single-item measure of EA. Zero participants demonstrated reduced EA as measured by the AAQ-II. Meanwhile, participant 1 demonstrated deterioration in daily single-item measures of stress and EA. Moreover, they observed increased stress as measured by the DASS-21 (moving into the extremely severe range) although this increase did not meet the criteria for reliable or clinically significant change. Despite individual improvement for some participants, visual analysis, quantitative analyses, and particularly meta-analysis across participant effect sizes—when taken as a whole—suggest that a single-session ACT intervention is an insufficient dosage of ACT to effect a change in stress and EA as experienced by people with IBD.

Previous studies of ACT interventions for IBD have suggested ACT to be an acceptable and potentially effective intervention for stress, depression, and anxiety [34,37]. However, these previous studies have delivered larger dosages of ACT (one full-day workshop and an eight-week ACT group, respectively) and included elements of social support through delivery via group formats. Moreover, a review published after the completion of study 1 suggested that single-session ACT interventions are highly acceptable, feasible, and potentially effective for a variety of chronic health conditions [92]. Of note, the modal hours of ACT delivered by the reviewed studies were five, with intervention lengths ranging from two to eight hours (relative to approximately two hours in study 1). In view of this, it is suggested that a greater dosage of ACT is necessary via a brief intervention.

In addition to dosage, a heavy emphasis on valued action is proposed to be an integral component of brief ACT interventions [46,47]. For example, focused acceptance and commitment therapy (FACT) is a brief variation of ACT typically employed in primary care health settings that heavily targets values and includes brief case conceptualization [47]. Indeed, Kroska et al. trialled brief FACT-informed interventions and observed that interventions 90 min and three hours in duration produced a reduction in depression comparable to a one-day workshop. As such, it is suggested that future ACT interventions for IBD not only include an expanded dosage but also FACT components such as FACT-based case conceptualization and greater emphasis on values.

The limitations of study 1 largely centre on the measures employed. Firstly, measures of EA may have been unable to accurately capture EA or the breadth of psychological flexibility. Regarding the AAQ-II, recent concerns have been raised regarding what it truly measures. Indeed, it is suggested that the AAQ-II may represent distress rather than EA or indices of psychological flexibility and inflexibility [89,90]. While the single-item measure of EA may have been adequate to capture the same, it is unlikely to capture the multifaceted construct of psychological flexibility well. Modern measures of psychological flexibility (such as CompACT) tend to map onto the ACT triflex by capturing openness to experience, behavioural and present moment awareness, and valued action [93]. It is suggested that single-item measures mirroring these aspects of the CompACT be included in study two. Moreover, in including a measure of valued action, it will be possible to assess change in functioning which is important in populations living with chronic health conditions and arguably more congruent with the a priori goals of ACT [24,92].

To conclude, study one suggests that a single-session ACT intervention of two hours in duration is largely insufficient to affect a reduction in stress, experiential avoidance, and other forms of distress for people with IBD. In response to the findings, interpretation, and limitations of study 1, study 2 employs a higher dosage ACT intervention which places greater emphasis on valued action and employs more valid and multifaceted measures of psychological flexibility.

## 4. Study 2: Randomized Multiple-Baseline Design Trial of a Two-Session Telehealth ACT Intervention

### 4.1. Study 2 Materials and Methods

#### 4.1.1. Participants

**Figure A2:**
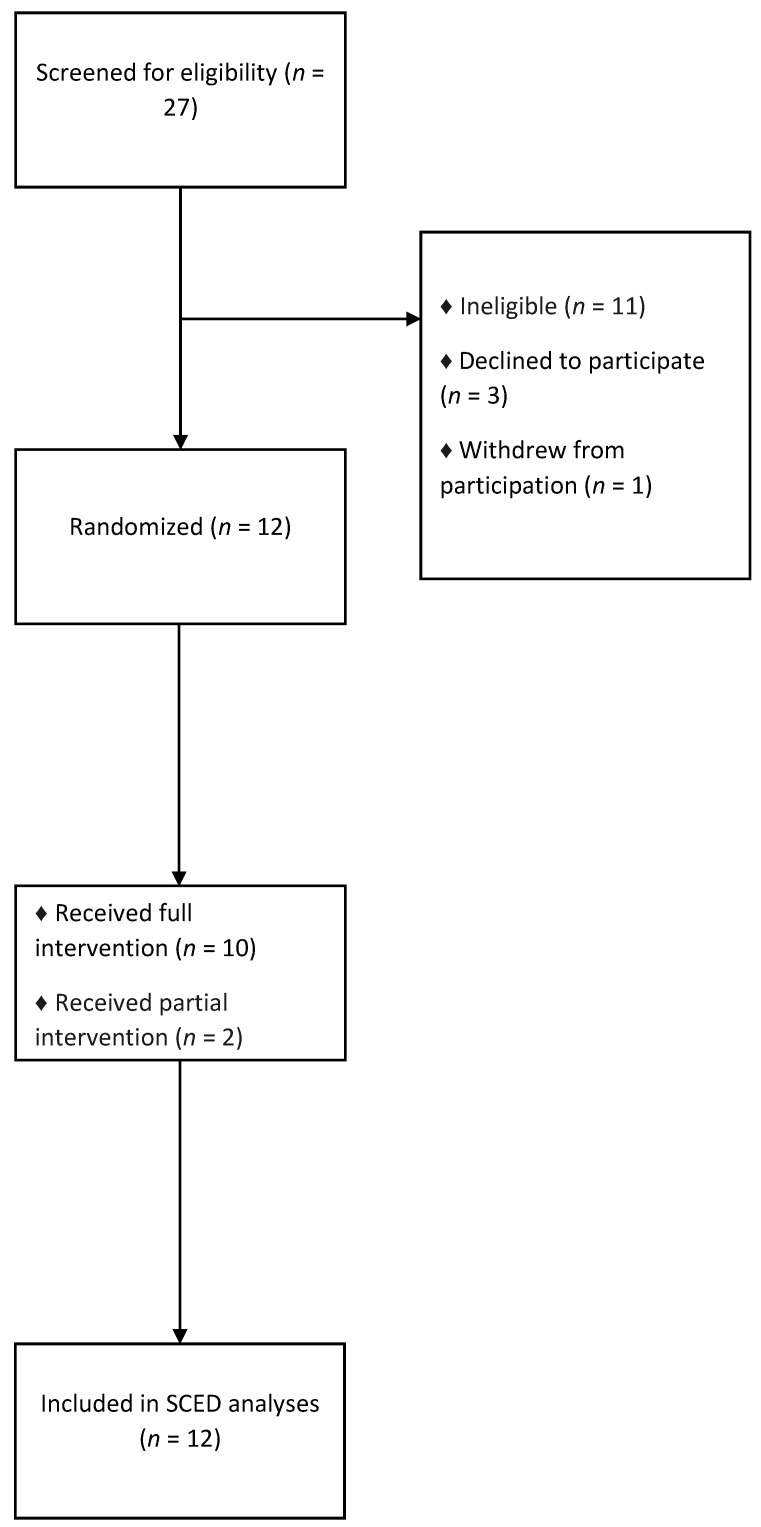
Study 2 participant flowchart.

The recruitment strategy described under Section 2.5 identified sixteen prospective participants. Three participants withdrew before the commencement of the study citing a return to in-office working, educational commitments, and a recent cancer diagnosis. One further participant provided informed consent but dropped out during the baseline assessment phase (i.e., before receiving the intervention) without providing any data or reasons for withdrawal. The final sample consisted of 12 participants scoring in the mild to the extremely severe range for stress on the DASS-21 (nine identifying as female, three identifying as male; *M* age = 36.42, *SD* = 11.16). Further participant details and baseline characteristics are available in Table 6. Recruitment is summarized in Figure A2 (Appendix A).

#### 4.1.2. Study Context: COVID-19 Pandemic

While the institutional context for study 2 remained the same as study 1, it is of note that the study was conducted during the COVID-19 pandemic. When the research commenced (4 May 2021), strict restrictions were in place to curb the spread of COVID-19. For example, work from home orders were in place, non-essential retail was prohibited from trading, individuals from different households were not permitted to socialize indoors, domestic and international travel were prohibited, etc. Commencing on 17 May 2021, these restrictions were lifted on a phased basis such that substantial limits to social, family, and working life had been removed by the conclusion of the study.

#### 4.1.3. Measures of Psychological Flexibility (PF)

Per the discussion of study 1 and limitations raised, alternative measures of PF were employed for study 2.

##### The Comprehensive Assessment of Acceptance and Commitment Therapy Processes (CompACT)

At the beginning and end of the study, the Comprehensive assessment of Acceptance and Commitment Therapy processes (CompACT) was administered as a measure of PF. The CompACT is a 23-item measure of PF which consists of three subscales, namely, openness to experience, behavioural awareness, and valued action. The CompACT takes approximately ten to fifteen minutes to complete. The CompACT is considered a reliable (internal consistency for the three subscales and total scale score were α = 0.90, α = 0.87, α = 0.90, and α = 0.91, respectively) and valid measure of psychological flexibility with factor analysis supporting the three-subscale factor structure [93,94]. Moreover, it is argued to be a more robust and valid measure of psychological flexibility relative to the extensively employed AAQ-II [87,89,90].

##### Daily Measures of PF—The Brief Acceptance Measure

The brief acceptance measure (BAM) is a SCED-oriented three-item measure of psychological flexibility including openness to experience, awareness, and valued action and typically takes up to three minutes to complete [95,96]. Each item incorporates a ten-point scale with higher scores denoting greater PF. The BAM has been validated for use in SCEDs and individual items and the overall score correlates with the corresponding components of the CompACT [95]. As presented in the present study, the BAM read as follows:

*This short scale asks how you have been today, please select a number anywhere along each scale, based on your own sense of which statement best describes how you have been. Today I have been…*

*(a) Struggling with my thoughts, feelings or physical sensations*

*1 2 3 4 5 6 7 8 9 10*

*Open to my thoughts, feelings, or physical sensations*

*(b) Acting without awareness*

*1 2 3 4 5 6 7 8 9 10*

*Acting with awareness*

*(c) Not pursuing things that matter to me*

*1 2 3 4 5 6 7 8 9 10*

*Pursuing things that matter to me*

#### 4.1.4. Measure of COVID-19-Related Stress: The COVID Stress Scales (CSS)

Given the potentially confounding impact of COVID-related distress, a measure of the same was included, namely the COVID Stress Scales (CSS). The CSS is a 36-item measure of COVID distress. The CSS asks respondents to rate the extent to which they have experienced a range of distress symptoms and phenomena on a scale of zero to four with a score of zero denoting “not at all” and a score of four denoting “extremely”. While the CSS is a novel measure, initial data suggest that the CSS is reliable (Cronbach’s α for individual subscales ranged from 0.86–0.95) and valid [97]. While an initial five-factor structure was proposed, subsequent studies have proposed a six-factor solution [97,98].

#### 4.1.5. ACT Adherence Quiz

A brief adherence measure was included to ensure participants understood the intervention. The adherence quiz included eleven multiple-choice items, each of which asked about the six core ACT processes individually and the overall key messages of the ACT intervention. One point was awarded per correct answer with possible scores ranging from 0 to 11. For example, one item checks the participants’ understanding of cognitive defusion, as follows (wherein the most appropriate response is d):

*“Fusion is:*

*(a) Thinking that our thoughts and reality are one and the same*

*(b) Seeing the world from your thoughts rather than observing your thoughts*

*(c) when stuck in our thoughts which tends to take us out of the present moment*

*(d) All of the above”*

#### 4.1.6. Two-Session Telehealth ACT Intervention

The intervention trialled under study two attempted to provide greater exposure to ACT or to administer a greater dosage of ACT to participants. The intervention consisted of three primary components:

1. Two telehealth intervention sessions were delivered by the first and third authors and under the supervision of the last author. Given the COVID-19 pandemic and accessibility, these sessions were delivered via the widely available video conferencing platform Zoom. Intervention sessions were typically two hours in duration (four total hours of contact time).
2. An ACT for IBD workbook.
3. A brief phone call follow-up consultation (conducted by the first and third authors, respectively, and typically lasting fifteen minutes).

##### Telehealth ACT Intervention Protocol

The telehealth ACT intervention protocol mirrored that trialled under study 1 and is summarized in Table 7. The intervention protocol was delivered across two sessions (each lasting approximately two hours) via the video conferencing platform Zoom. Session one focused on providing psychoeducation on ACT, IBD, and the relationship between stress and disease activity and severity in IBD. A notable addition was the inclusion of information gathering and case conceptualization via focusing questions derived from focused acceptance and commitment therapy (FACT) [46,47]. These were employed to better understand participants presenting concerns and, expectations, and to better apply subsequent intervention components to their contextual needs. The remainder of session one focused on introducing acceptance strategies to promote openness to internal experiences and mirrored phases four and five described under Section 3.1.3.

Session two occurred approximately seven days later and commenced with an exercise designed to ground the participant and promote engagement in the intervention session ahead. The ACT matrix exercise was repurposed in study 2 and employed as a case conceptualization tool. At the start of session 2, the interventionist summarized the information gathered and content covered in session 1 using the ACT matrix to facilitate this. Participants were subsequently guided in an exercise to promote compassionately responding to oneself (mirroring phase 7; Section 3.1.3). Given the remote delivery format, values clarification was assisted via a values checklist which asked participants to rate the personal importance of a selection of commonly held values. Lastly, the ACT matrix used in phase six of the intervention trialled under study 1 was replaced by a goal-setting worksheet which was deemed to be more suitable and acceptable for the remote intervention format. Said exercise (derived from Wynne et al.), supported participants in creating short-, medium-, and long-term goals that reflected the values identified during the previous phase. Moreover, barriers to the successful completion of these goals were identified, as was an action plan to employ skills taught as part of the intervention in response to internal barriers.

##### ACT for IBD Workbook

Participants were provided an ACT for IBD workbook adapted for study 2 from established ACT protocols and workbooks for long-term health conditions [37,99,100]. The workbook facilitated the further practice of ACT processes introduced during intervention sessions and included additional elements considered helpful for living effectively with long-term health conditions: problem-solving skills, self-pacing, and assertive communication. To enhance acceptability and relevance, specific examples were included about IBD. For example, common thoughts one might experience while living with IBD. Chapters one and two were assigned to be completed after session one of the intervention and centred on acceptance, defusion, and values. The remaining chapters were assigned after session two and focused on problem-solving, effective communication, and goal-setting.

##### Phone Follow-Up Consultation

A brief phone follow-up consultation occurred fourteen days after the second intervention session and was facilitated by the interventionist who had delivered the participant’s intervention sessions. This component was modelled on existing brief interventions supplemented by phone follow-ups [101]. This component lasted approximately fifteen minutes and was designed to promote accountability. Troubleshooting of the ACT process and skill practice was also facilitated.

#### 4.1.7. Procedure

The procedure mirrored that detailed under Section 3.1.4. Participants who provided informed consent completed the DASS-21, CompACT, and CSS on week 0 (immediately before the study commencement) and post-study. From days 1 to 101 participants provided daily reports of stress and PF via the single-item measures described under Section 2.6.2 and Section “Daily Measures of PF—The Brief Acceptance Measure”, respectively. Single-item measures of stress and PF were delivered via a single SMS message at 6:00 p.m. GMT daily and sent using a research phone operated by the first author. Participants were encouraged to provide labelled responses via a single SMS message reply.

From day 21 onwards, participants began to receive the two-session telehealth ACT intervention per the randomized order. Intervention sessions were scheduled at a time amenable to the participant by the first author. Intervention components were delivered by the first and third authors per the protocol outlined under Section 4.1.6.

### 4.2. Study 2 Results

#### 4.2.1. Missing Data

Missing data on daily measures of stress and PF ranged from 0% to approximately 35% and are summarized in Table 8. Five participants (participants 3, 4, 5, 11 and 12) did not complete the DASS-21, CompACT, or CSS post-study.

#### 4.2.2. Treatment Adherence and Protocol Compliance

Treatment adherence is summarized in Table 9. Table 9 also notes individual completion of intervention components, the number of missed intervention sessions (DNAs), the number of rescheduled sessions, the order in which the participant was randomized to receive intervention, and the order in which they received the intervention. Participants 5 and 7 did not receive all intervention components by their own choice, citing work and family commitments in both cases. The randomized order was compromised on occasion to ensure the timely completion of the study and minimize the assessment burden on participants. Reasons for the same included non-attendance of intervention sessions (see DNA and rescheduled sessions, Table 9) and hospitalization.

#### 4.2.3. Stress and PF as Measured by Daily Items

**Figure A3:**
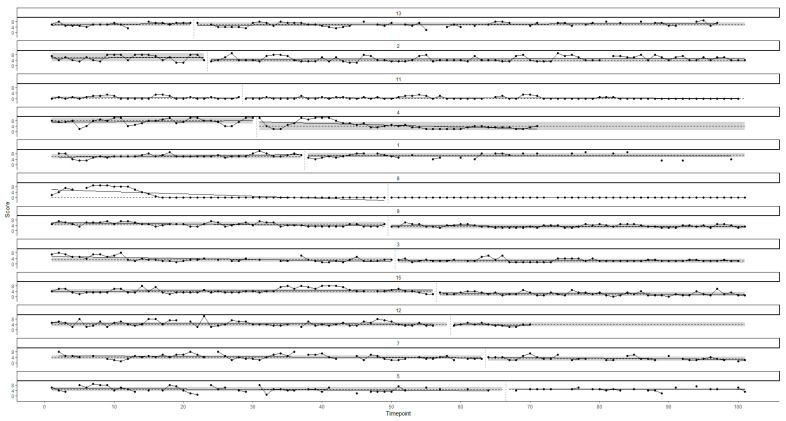
SCED data for all study 2 participants on daily measure of stress. Note: Participant data are ordered via the order in which they received the intervention. Vertical dotted lines denote the data point at which the intervention was delivered. Shading denotes the one median absolute deviation from the median. Dashed horizontal lines denote the median while solid lines denote within-phase OLS regression trend lines.

**Figure A4:**
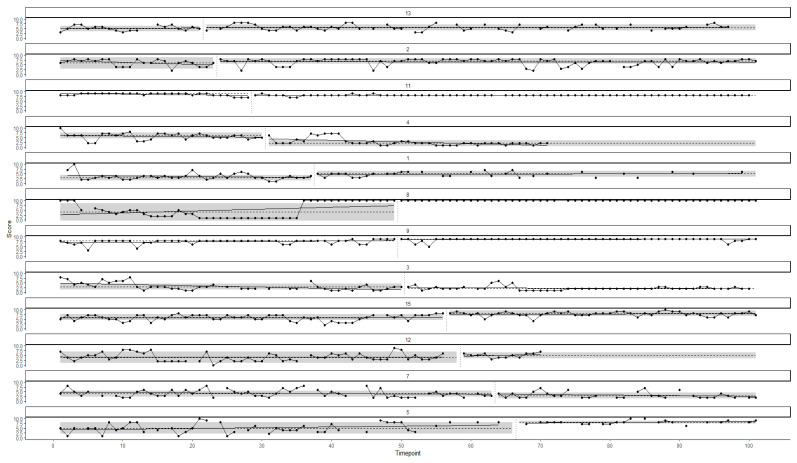
SCED data for all study 2 participants on daily measure of openness to experience. Note: Participant data are ordered via the order in which they received the intervention. Vertical dotted lines denote the data point at which the intervention was delivered. Shading denotes the one median absolute deviation from the median. Dashed horizontal lines denote the median while solid lines denote within-phase OLS regression trend lines.

**Figure A5:**
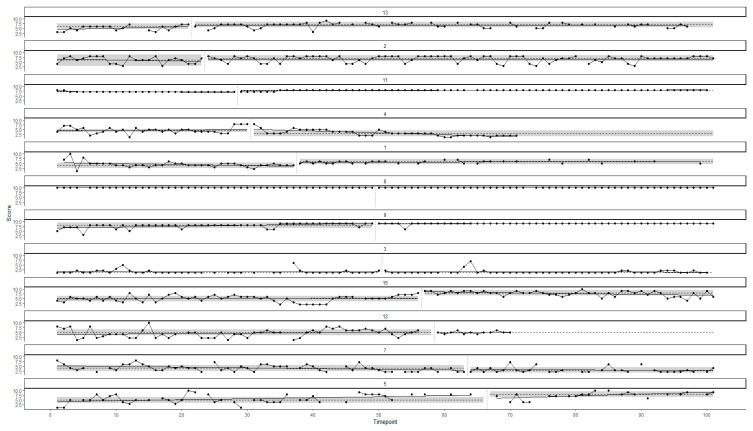
SCED data for all study 2 participants on daily measure of awareness. Note: Participant data are ordered via the order in which they received the intervention. Vertical dotted lines denote the data point at which the intervention was delivered. Shading denotes the one median absolute deviation from the median. Dashed horizontal lines denote the median while solid lines denote within-phase OLS regression trend lines.

**Figure A6:**
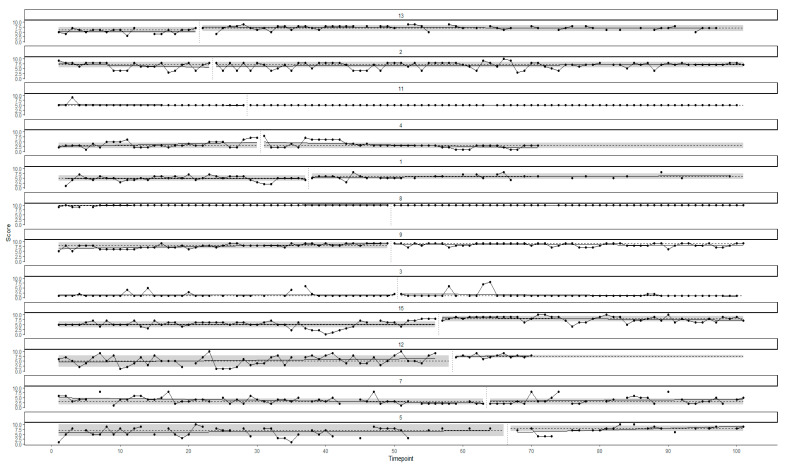
SCED data for all participants on daily measure of valued action. Note: Participant data are ordered via the order in which they received the intervention. Vertical dotted lines denote the data point at which the intervention was delivered. Shading denotes the one median absolute deviation from the median. Dashed horizontal lines denote the median while solid lines denote within-phase OLS regression trend lines.

Data for stress and PF variables are presented in Figure A3, Figure A4, Figure A5 and Figure A6 (see Appendix A). The plot orders participant data per the order in which they received the ACT intervention in order to visualize the impact of the intervention across multiple baselines. Following visual inspection of the plots, four participants (see Figure A3; participants 2, 4, 9, and 15) appeared to demonstrate reduced stress following exposure to the ACT intervention. Four participants appeared to show increased openness to experience (see Figure A4; participants 1, 2, 12, and 15).

Five participants (see Figure A5; participants 1, 2, 5, 13, and 15) appeared to demonstrate increased awareness following exposure to the ACT intervention. Four participants (see Figure A6; participants 1, 5, 12, and 15) appeared to display increased valued action following the ACT intervention.

Following quantitative analyses of stress data, it was observed that 6 of 12 participants (see Table 10; participants 3, 4, 7, 8, 9, and 15; 50%) demonstrated reduced stress following exposure to the ACT intervention. Given the presence of baseline trends which could indicate natural improvement, participants demonstrating baseline OLS regressions trends of ±0.3 were excluded from the meta-analysis across participants. The meta-analysis was significant, indicating reduced stress as a function of the ACT intervention, *N* = 7, unstandardized effect size (median median-difference) = −1, *P*(A < B) = 0.331, 95% CI [0.290, 0.422], 95% CR [0.182, 0.573], *p* < 0.0001, generalized OR = 0.55, 95% CI [0.41, 0.73], 95% CR [0.22, 0.73].

Following quantitative analyses of openness to experience data, it was observed that 5 of 12 participants (see Table 10; participants 1, 5, 8, 9, and 15; 42%) demonstrated increased openness to experience following exposure to the ACT intervention. However, 4 of 12 participants (participants 3, 4, 7, and 11; 33.33%) displayed reduced openness to experience following the ACT intervention. The meta-analysis, with baseline trends excluded, was not significant, indicating the absence of a generalizable effect on openness to experience, *N* = 8, unstandardized effect size (median median-difference) = 1.5, *P*(A < B) = 0.635, 95% CI [0.426, 0.803], 95% CR [0.127, 0.954], *p* = 0.2025, generalized OR = 1.74, 95% CI [0.74, 4.06], 95% CR [0.15, 4.06].

Analyses of awareness data suggest that 6 of 12 participants (see Table 11; participants 1, 5, 9, 11, 13, and 15; 50%) demonstrated increased awareness as a function of the ACT intervention. Of 12 participants, 2 (participants 4 and 7; 17%) saw reduced awareness following exposure to the ACT intervention. A meta-analysis with participants exhibiting baseline trends excluded suggested an absence of generalizable treatment effects, *N* = 7, unstandardized effect size (median median-difference) = 1, *P*(A < B) = 0.686, 95% CI [0.460, 0.848], 95% CR [0.141, 0.967], *p* = 0.1039, generalized OR = 2.18, 95% CI [0.85, 5.58], 95% CR [0.16, 5.58].

Following quantitative analyses of valued action data, it was observed that 7 of 12 participants (see Table 11; participants 1, 5, 8, 9, 12, 13, and 15; 58%) demonstrated increased valued action following exposure to the ACT intervention. Meta-analysis of participant effect sizes suggested a generalizable improvement in valued action, *N* = 7, unstandardized effect size (median median-difference) = 1, *P*(A < B) = 0.711, 95% CI [0.571, 0.820], 95% CR [0.320, 0.928], *p* = 0.0041, generalized OR = 2.46, 95% CI [1.33, 4.55], 95% CR [0.47, 4.55].

#### 4.2.4. DASS-21 Measured Depression, Anxiety, and Stress

Seven participants completed the DASS-21 at both time points (see Table 12). Three participants (participants 1, 2, and 15) demonstrated reliable improvements in stress with one meeting the criteria for clinically significant change (participant 15). Zero participants made reliable or clinically significant improvements in depression. Participant 2 demonstrated a reliable improvement in anxiety that was not clinically significant. Zero participants demonstrated deteriorations in DASS-21 measured depression, anxiety, or stress that were reliable or clinically significant.

#### 4.2.5. CompACT-Measured PF

CompACT-measured PF was reported by seven participants at both time points and is reported in Table 13 below. In lieu of appropriate clinical norms for the CompACT, it was not possible to assess clinically significant change. Reliable improvements or deteriorations were not observed on the CompACT or its subscales for any participants.

#### 4.2.6. CSS-Measured COVID Stress

Seven participants completed the CSS at both time points. In lieu of suitable clinical and comparison norms, it was not possible to assess reliable or clinically significant change. While six participants reported reduced COVID-19-related stress at time two (see Table 13), it is suggested this is a function of the changing COVID-19 situation in Ireland during the trial rather than a function of the intervention.

## 5. Discussion

Study 2 employed a randomized multiple baseline design to investigate the effectiveness of a two-session telehealth ACT intervention supplemented by an ACT-based workbook and phone call consultation for IBD. It was predicted that the ACT intervention would produce reductions in stress and promote increased engagement in openness to internal experiences, awareness, and valued action. At the individual level, 6 of 12 participants demonstrated reduced stress (participants 3, 4, 7, 8, 9, and 15; 50%), 5 of 12 demonstrated increased openness to experience (participants 1, 2, 5, 13, and 15; 42%), 6 of 12 demonstrated increased awareness (participants 1, 5, 9, 11, 13, and 15; 50%), and 7 of 12 demonstrated increased engagement in valued action (participants 1, 5, 8, 9, 12, 13, and 15; 58%) on single-item measures of stress and PF. Moreover, three participants (participants 1, 2, and 15) demonstrated reliable improvements in DASS-21 measured stress (which was clinically significant for participant 15). Participant two demonstrated a reliable improvement in anxiety. Neither reliable nor clinically significant changes were evident in DASS-21-measured depression, and facets of CompACT-measured PF. Deteriorations were evident for 4 of 12 participants (participants 3, 4, 7, and 11; 33.33%) and 2 of 12 participants (participants 4 and 7; 17%) on single-item-measured openness to experience and awareness, respectively. However, findings must be approached with caution given significant missing data for some participants (participants 1, 4, 12, and 13) and the minimal variation in participant 8’s data. Meta-analysis across participants suggested reductions in stress and increased valued action as a function of the intervention.

Improvements in openness to experience, awareness, and particularly stress and valued action are broadly congruent with the existing literature on brief interventions and improvements early in therapy. For example, sudden improvements for 40 to 60% of clients following brief interventions and initial therapy sessions are a well-observed effect in the cognitive behavioural therapies [41,42,43,44]. While ostensibly used as a measure of valued action, the single-item measure of the same also functioned as a proxy for functioning. Given this, we suggest that the intervention functioned to decrease stress and increase functioning and is congruent with previous research on brief ACT interventions [34,35,92]. Moreover, these findings seem conceptually congruent with findings linking increased valued action (and reduced avoidance functioning behaviour) with improved psychological health and functioning in IBD and chronic conditions [33,100,102,103].

Despite between 40% and 58% of participants experiencing improvements across various stress and psychological flexibility outcomes, many participants experienced minimal change or saw deterioration following exposure to the ACT intervention. While assumed to be exclusively beneficial, psychological, and behavioural therapies may on occasion confer deterioration and harm [104,105,106]. While the present study is not well placed to ascertain factors and processes associated with improvement and deterioration, we note that adherence to treatment protocol and content may have influenced deterioration. That is, for some participants deterioration occurred in context of poorer adherence or not having engaged in all intervention components. Regarding the absence of changes, we note that in some cases change may have been obfuscated by that participant’s dropout. That is, treatment effects may have emerged had the participant continued to report data throughout the study period. In other cases, participants experienced improvement between screening and the start of the study, potentially causing a floor effect. For example, participant 11 presented at the start of the study with higher psychological flexibility relative to most other participants and had moved into the normal range for stress (i.e., had met inclusion criteria at screening but no longer met the same at the study’s start). Lastly, we note that the presence of baseline improvement trends may, on occasion, have obfuscated improvement and deterioration as a function of the intervention. Such trends may follow from assessment wherein assessment functions as an intervention. For example, reporting low engagement in valued action may elicit dissonance for an individual and motivate greater engagement in valued action on the subsequent day(s) [107,108]. While ineffectiveness and deterioration occurred following the intervention, we argue that adherence to protocol, dropout, and improvement trends in response to assessment as plausible and competing explanations for these effects being the function of the ACT intervention.

While approximately half of the participants experienced improvements in stress and valued action, these trends were not reliably matched by reliable and clinically significant changes in DASS-21-measured stress and CompACT-measured PF. This discordance between daily single-item indices of stress and PF and established measures of the same suggests that changes in single-item measures were of a small magnitude. While changes for some participants were statistically significant, these changes may not be associated with clinically meaningful change. However, it could be argued that a change of this magnitude such that participants’ scores fall into the range of healthy peers may be unreasonable given the persistence and impact of lifelong illness in the form of IBD [80]. Moreover, a change of a smaller magnitude may still confer personally meaningful benefits and facilitate more purposeful and vital living in the context of long-term illness.

### 5.1. Strengths and Limitations

The strengths of the present studies centre on the design used and the approach to data sampling. Firstly, single-case experimental designs facilitated the investigation of two novel and brief ACT interventions. Given the novel nature of the interventions and the paucity of evidence for their use in populations of people with IBD, it was important to be enabled to assess both the benefit and risk conferred by exposure to the brief interventions. Such investigations (especially of risk) are typically not feasible via nomothetic group-based designs [55]. Moreover, the use of concurrent baselines minimized the effect of factors such as the passage of time and maturation [109]. Lastly, the collection of daily self-reported stress, EA, and PF at specified times (i.e., ecological momentary assessment) increased ecological validity and minimized biases such as recall bias which may impact other types of self-report measures [50]. Such phenomena and the variables of study were measured as they occurred or in close temporal proximity to their occurrence rather than retrospectively (as is the case with measures such as the DASS-21).

A limitation of the present study was the absence of a comparator intervention. As such, improvement may simply reflect the receipt of an intervention rather than the active components of the intervention being studied. However, we argue that the findings of study 1 attenuate this limitation somewhat given that the delivery of an intervention, in that case, did not convey significant improvement. A second limitation was that existing psychological diagnoses and the receipt of psychological support were not screened for. No participants disclosed existing conditions or the receipt of psychological therapy (e.g., via demographic questionnaires or to their interventionist); however, some may have chosen not to disclose this information. As such, some effectiveness or lack thereof of the interventions trialled under studies 1 and 2 may be attributable to concurrent therapies and undisclosed mental health conditions. While not a limitation per se, an important caveat to the findings of study 2 was the evolving COVID-19 situation. As noted under Section 4.1.2, some improvement may have been attributable to the easing of restrictions on social and economic life.

A further limitation centres on the generalizability of findings. While adequately powered for SCED methodology and employing a relatively large number of participants and data points (many SCEDs include three or fewer participants and three datapoints per phase), the samples for studies 1 and 2 remain relatively small and homogenous (participants were predominantly young adults and female) [83,84]. While the meta-analysis of effects across participants enhances generalizability somewhat, larger studies and further replications are necessary to afford greater generalizability. Moreover, generalizability and replicability may be hampered by the single-item measures used. At present, little consensus exists with respect to measurement in SCEDs, which impedes the comparison and generalizability of findings [51]. As such, SCEDs may study the same or similar outcomes using different measures of outcomes, hampering the generalizability and synthesis of findings.

### 5.2. Implications and Future Research

The present studies lend further evidence that brief, telehealth ACT interventions with blended delivery components including workbooks and phone consultations are feasible for people with long-term health conditions and, specifically, IBD. Moreover, the findings of study 2 suggest such brief, blended and remote interventions to be potentially efficacious.

The present study saw interventions delivered by interventionists with training in ACT and counselling and clinical skills. Interventionists, however, did not have recognized professional training in clinical psychology, psychotherapy, or counselling. As such, it may be feasible for psychology graduates or other allied health professionals to deliver this intervention (or similar interventions) within healthcare services catering to people with IBD. As such, future research should investigate the feasibility and efficacy of this approach to intervention delivery given the potential for improved access to psychological therapies for people with IBD.

Future research should replicate the methodology of the present studies and investigate the acceptability and efficacy of these and other blended components. For example, other adjuncts might include digital self-help programs, bibliotherapy, or chatbot-delivered interventions. Lastly, further research is necessary to elucidate factors that influence suitability for brief interventions. As observed in study 2, approximately 40% to 58% of people with IBD may benefit from a brief ACT intervention. Future research is necessary to better identify those who may benefit most to facilitate the prescription of the same.

## 6. Conclusions

Although attenuated by concerns around missing data and variation in the data, the context of COVID-19, and measures of experiential avoidance, the present findings provide insight on appropriate dosages of ACT to effect changes in stress and psychological flexibility in people with IBD. While seemingly feasible, it appears that little benefit is conferred via a single-session ACT intervention. Meanwhile, a two-session telehealth ACT intervention supplemented via an ACT workbook and follow-up phone call appears to be somewhat effective for people with IBD. However, further replication and study is warranted including triaging those best suited for such interventions.

## Figures and Tables

**Table 1:** Participant gender, IBD diagnosis, and DASS-measured stress at study commencement.

Participant ID	Gender	IBD Diagnosis	DASS-21 Stress	Comorbid Diagnoses	Medical/Surgical Treatment	Last IBD Flare
1	Female	Crohn’s Disease	Severe	No	Medicine only ^1^	09/2019
4	Female	Ulcerative Colitis	Moderate	No	Medicine only	10/2019 (ongoing) ^2^
5	Male	Ulcerative Colitis	Severe	No	Medicine only	01/2018
6	Male	Ulcerative Colitis	Moderate	No	Medicine only	10/2019 (ongoing)
7	Female	Ulcerative Colitis	Moderate	IBS	Medicine only	06/2019
8	Female	Crohn’s Disease	Extreme	No	Medicine only	08/2019 (ongoing)
10	Female	Crohn’s Disease	Severe	No	Medicine only	07/2019

Note: ^1^ Participant received medical intervention (typically steroidal or biologic treatments) to manage IBD but had not at that time received surgical intervention. ^2^ Participant’s disease flare was ongoing during their participation in the study (October–December 2019).

**Table 2:** Summary of single-session ACT intervention protocol.

Phase	Aims	Exercise
Psychoeducation (10 min)	To introduce the link between stress and disease activity in IBD	Description of stress and IBD
Creative hopelessness (15 min)	To introduce the unworkability of control strategies	Polygraph metaphor
		Attempts and evaluations worksheet
Defusion (20 min)	To notice thoughts as barriers to action	Content on cards
		Taking your mind for a walk
		I’m having the thought that…
Acceptance (15 min)	To develop willingness to experience internal events (such as stress)	Physicalizing mindfulness exercise
Values (20 min)	To clarify personally meaningful values for the participant	Values cards sort
Committed action (20 min)	To encourage workable behaviour change	Matrix
Self-compassion (20 min)	To promote self-compassion	Compassionate hand

**Table 3:** SCED quantitative analyses summary for stress and experiential avoidance.

Participant	Stress					EA				
	Trend	Median	*p*	Ruscio’s A	Hedges *g*	Trend	Median	*p*	Ruscio’s A	Hedges *g*
1	0.01	2.0	0.0028	0.733	0.85	0.00	2.0	0.0012	0.740	0.90
4	−0.01	0.0	0.4195	0.561	0.25	0.00	0.0	0.7940	0.479	−0.09
5	−0.50	0.0	0.7964	0.520	−0.09	−0.56	1.0	0.4801	0.583	0.21
6	0.23	−1.5	0.0175	0.328	−0.68	0.05	0.0	0.8577	0.492	−0.06
7	−0.29	0.5	0.4135	0.581	0.25	−0.20	0.0	0.0442	0.470	−0.60
8	0.48	−1.0	0.6147	0.423	−0.15	0.31	−1.0	0.0003	0.230	−1.13
10	−0.12	−2.0	0.2948	0.429	−0.28	−0.06	−1.5	0.0107	0.295	−0.69

Note: Trend denotes OLS regression trend during phase A (i.e., before intervention); median denotes the median difference between phases.

**Table 4:** DASS-21 measured depression, anxiety, and stress at pre- and post-study.

Participant ID	Time 1						Time 2					
	Depression		Anxiety		Stress		Depression		Anxiety		Stress	
1	Moderate	20	Moderate	14	Severe	30	Extreme	30	Extreme	26	Extreme	34
4	Mild	10	Extreme	22	Moderate	24	-	-	-	-	-	-
5	Normal	2	Moderate	12	Severe	32	-	-	-	-	-	-
6	Normal	2	Normal	4	Moderate	24	Normal	2	Moderate	12	Moderate	20
7	Normal	8	Severe	18	Moderate	22	Normal	0	Normal	4 **	Normal	10 **
8	Moderate	18	Extreme	32	Extreme	38	Normal	6 **	Mild	10 **	Mild	16 *
10	Moderate	14	Mild	8	Severe	32	Moderate	18	Normal	2	Mild	18 *

Note: Severity bands are derived from the DASS-21 manual and appear to the left of the participants’ scores [62]. Extreme denotes a severity band of extremely severe. * Denotes that reliable change was observed; ** denotes that both reliable and clinically significant change were observed.

**Table 5:** Participant AAQ-II scores at times one and two.

Participant ID	Time 1 AAQ-II	Time 2 AAQ-II	Change
1	29	31	+2
4	25		
5	25		
6	28	22	−6
7	19	17	−2
8	26	23	−3
10	44	41	−3

**Table 6:** Participant gender, IBD diagnosis, and DASS-measured stress at screening.

Participant ID	Gender	IBD Diagnosis	DASS-21 Stress at Screening	Comorbid Diagnoses	Medical/Surgical Treatment	Most Recent Disease Flare
1	Female	Indeterminate Colitis	Severe	No	Medicine only ^1^	05/2021 (Ongoing) ^2^
2	Female	Ulcerative Colitis	Severe	No	Medicine only	02/2021
3	Female	Crohn’s Disease	Extreme	No	Medicine only	04/2021 (Ongoing)
4	Female	Crohn’s Disease	Moderate	No	Medicine only	01/2021 (ongoing)
5	Female	Crohn’s Disease	Mild	No	Medicine only	02/2020
7	Female	Crohn’s Disease	Extreme	No	Medicine only	11/2020
8	Female	Ulcerative Colitis	Moderate	Diabetes (Type 1)	Medicine only	01/2021
9	Male	Ulcerative Colitis	Moderate	No	Medicine only	02/2020
11	Male	Ulcerative Colitis	Mild	No	Medicine only	2018
12	Male	Indeterminate Colitis	Severe	No	Medicine only	02/2021
13	Female	Ulcerative Colitis	Severe	No	Medicine only	09/2020
15	Female	Crohn’s Disease	Severe	Gastritis; Diverticulosis	Medicine only	07/2018

Note: ^1^ Participant received medical intervention (typically steroidal or biologic therapies) to manage IBD but had not at that time received surgical intervention (e.g., resection). ^2^ Participant’s disease flare was ongoing during their participation in the study (May–August 2021).

**Table 7:** Two-session telehealth ACT intervention protocol.

Session	Phase	Aims	Exercise
1	Introduction and psychoeducation (10 min)	To introduce the structure of intervention and the link between stress and disease activity in IBD	Description of stress and IBD
	Case conceptualization (35 min)	To gather information and conduct case formulization	Focusing questions (FACT)
			Attempts and evaluations worksheet
	Creative hopelessness (15 min)	To further elaborate on control as the problem	Polygraph metaphor
	Defusion (20 min)	To notice thoughts as barriers to action	Content on cards
			Taking your mind for a walk
			I’m having the thought that…
	Acceptance (15 min)	To develop willingness to experience internal events	Physicalizing mindfulness exercise
	Committed action and homework (10 min)	To set a SMART goal based on what has been identified as important via focusing questions and assign homework	
2	Check-in (5 min)	To check on the progress of the SMART goal set at end of session one and review homework	
	Intention setting/grounding exercise (10 min)	To promote present-moment awareness and promote awareness of toward and away moves in vivo	
	Review of previous session (10 min)	To re-cap on processes introduced in previous session and present information from session one in an ACT-consistent way	Matrix
	Self-compassion (20 min)	To promote self-compassion	Compassionate hand
	Values (20 min)	To identify and clarify values that are personally meaningful to the participant	Values clarification worksheet
	Committed action (20 min)	To encourage workable behaviour change	SMART Goal Setting worksheet
	Homework (5 min)	To set homework and arrange a time for phone follow-up	

**Table 8:** Missing data per participant on daily measures of stress and PF.

ID	Total		Phase A			Phase B		
	Total Missed Sessions	Percentage Missing	Missed Sessions	Total Sessions	Missing Percent	Missed Sessions	Total Sessions	Missing Percent
1	34	33.70%	1	37	02.70%	33	64	51.56%
2	1	01.00%	0	23	00.00%	1	78	01.28%
3	8	07.90%	6	50	12.00%	2	51	03.92%
4	30	29.07%	0	30	00.00%	30	71	42.25%
5	34	33.70%	21	66	31.80%	13	35	37.14%
7	16	15.80%	9	63	14.28%	7	38	18.42%
8	2	01.90%	1	49	02.04%	1	52	01.92%
9	0	00.00%	0	49	00.00%	0	52	00.00%
11	2	01.90%	0	28	00.00%	2	83	02.41%
12	35	34.70%	4	58	06.90%	31	43	72.09%
13	26	25.70%	2	21	09.52%	24	80	30.00%
15	1	00.90%	1	56	01.79%	0	45	00.00%

Note: Sessions and missed sessions denote daily stress and PF recording sessions that were missed by the participant.

**Table 9:** Treatment adherence and protocol compliance.

ID	Adherence Score (0–11)	Session 1	Session 2	Phone Consultation	DNA	Rescheduled Sessions	Interventionist	Randomized Order	Actual Order
1	5	Yes	Yes	Yes	1	0	Third	6	5
2	7	Yes	Yes	Yes	0	0	First	3	2
3	-	Yes	Yes	Yes	0	3	Third	4	8
4	-	Yes	Yes	Yes	1	1	First	5	4
5	-	Yes	No	No	0	1	First	12	12
7	3	Yes	No	No	2	0	Third	10	11
8	1	Yes	Yes	Yes	0	0	First	7	6
9	2	Yes	Yes	Yes	0	0	Third	9	7
11	-	Yes	Yes	Yes	2	0	First	1	3
12	-	Yes	Yes	Yes	1	0	First	11	10
13	8	Yes	Yes	Yes	0	0	First	2	1
15	3	Yes	Yes	Yes	0	1	First	8	9

Note: DNA denotes that the participant missed an intervention session without giving prior notification. Interventionist denotes which author (first or third) delivered intervention components.

**Table 10:** Quantitative analyses summaries for stress and openness to experience.

Participant	Stress					Openness				
	Trend	Median	*p*	Ruscio’s A	Hedges *g*	Trend	Median	*p*	Ruscio’s A	Hedges *g*
1	0.33 *	1.0	0.9391	0.505	−0.02	−0.05	2.0	0.0037	0.748	0.74
2	0.09	−2.0	0.1472	0.399	−0.36	−0.42 *	1.0	0.2742	0.598	0.26
3	−0.58 *	−1.0	0.0034	0.347	−0.63	−0.55 *	−1.0	0.0056	0.361	−0.59
4	0.15	−4.0	0.0001	0.207	−1.16	−0.22	−4.0	<0.0001	0.139	−1.63
5	−0.27	0.0	0.9410	0.523	−0.03	0.21	3.0	<0.0001	0.820	1.35
7	−0.26	−1.0	0.0070	0.322	−0.63	−0.18	−1.0	0.0174	0.333	−0.55
8	−0.65 *	0.0	0.0001	0.344	−0.85	0.22	6.0	<0.0001	0.823	1.79
9	−0.50 *	−2.0	0.0001	0.264	−0.95	0.31 *	1.0	<0.0001	0.878	1.17
11	−0.15	0.0	0.1150	0.409	−0.37	−0.34 *	−1.0	<0.0001	0.263	−1.12
12	−0.11	0.0	0.0828	0.358	−0.57	0.00	1.0	0.1680	0.660	0.45
13	0.42 *	0.0	0.7891	0.485	−0.08	0.01	0.5	0.2573	0.580	0.31
15	0.21	−2.0	0.0001	0.159	−1.36	0.08	2.0	<0.0001	0.848	1.40

Note: Trend denotes OLS regression trend during phase A (i.e., before intervention); median denotes the median difference between phases. * denotes baseline trends of ±0.3.

**Table 11:** Quantitative analyses summaries for awareness and valued action.

Participant	Stress					Openness				
	Trend	Median	*p*	Ruscio’s A	Hedges *g*	Trend	Median	*p*	Ruscio’s A	Hedges *g*
1	−0.29	2.0	<0.0001	0.842	1.01	0.00	1.0	0.0031	0.691	0.75
2	−0.24	1.0	0.1378	0.612	0.36	−0.34 *	0.0	0.6686	0.511	0.11
3	−0.11	0.0	0.7678	0.476	−0.08	0.02	0.0	0.9389	0.472	−0.02
4	0.02	−1.5	0.0006	0.252	−0.91	0.34 *	0.0	0.4306	0.448	−0.20
5	0.34 *	3.0	0.0012	0.719	0.87	0.06	1.0	0.0350	0.641	0.56
7	−0.38 *	−1.0	0.0052	0.310	−0.64	−0.52 *	0.0	0.8516	0.495	0.05
8	0.00 *	0.0	-	-	-	0.22	0.0	0.0486	0.542	0.43
9	0.65 *	1.0	<0.0001	0.843	1.21	0.67 *	1.0	0.0018	0.666	0.66
11	−0.20	1.0	<0.0001	0.922	3.04	−0.09	0.0	0.2859	0.482	−0.36
12	0.21	0.0	0.6358	0.548	0.17	0.21	2.5	0.0043	0.767	0.95
13	0.43 *	1.0	<0.0001	0.786	1.14	0.09	1.0	<0.0001	0.870	1.62
15	0.12	3.0	<0.0001	0.880	1.67	0.12	3.0	<0.0001	0.887	1.67

Note: Trend denotes OLS regression trend during phase A (i.e., before intervention); median denotes the median difference between phases. It was not possible to compute some test statistics via the R SCED package for participant 8 due to a complete lack of variability in their awareness data. * denotes baseline trends of ±0.3.

**Table 12:** DASS-21 measured depression, anxiety, and stress at pre- and post-study.

ID	Stress				Depression				Anxiety			
	Time 1		Time 2		Time 1		Time 2		Time 1		Time 2	
1	30	Severe	* 18	Mild	16	Moderate	20	Moderate	20	Extreme	14	Moderate
2	36	Extreme	* 20	Moderate	20	Moderate	12	Mild	30	Extreme	* 14	Moderate
3	24	Moderate			20	Moderate			28	Extreme		
4	16	Mild			8	Normal			16	Severe		
5	24	Moderate			4	Normal			2	Normal		
7	24	Moderate	32	Severe	10	Mild	14	Moderate	14	Moderate	20	Extreme
8	0	Normal	0	Normal	0	Normal	0	Normal	0	Normal	0	Normal
9	12	Normal	6	Normal	8	Normal	0	Normal	4	Normal	0	Normal
11	10	Normal			0	Normal			4	Normal		
12	22	Moderate			30	Extreme			10	Moderate		
13	24	Moderate	24	Moderate	18	Moderate	12	Mild	2	Normal	6	Normal
15	22	Moderate	** 8	Normal	16	Moderate	6	Normal	6	Normal	2	Normal

Note: Severity bands are derived from the DASS-21 manual and appear to the right of the participants’ scores [62]. Extreme denotes a severity band of extremely severe. * Denotes that reliable change was observed; ** denotes that both reliable and clinically significant change were observed.

**Table 13:** CompACT-measured PF and CSS COVID stress pre- and post-study.

ID		OE	Change		BA	Change		VA	Change		CompACT Total	Change		CSS Total	Change
	T1	T2		T1	T2		T1	T2		T1	T2		T1	T2	
1	7	12	+5	2	7	+5	37	34	−3	46	53	+7	40	8	−32
2	26	27	+1	7	8	+1	27	24	−3	60	59	−1	85	40	−45
3	17			13			38			68			27		
4	41			22			3			66			26		
5	29			22			34			85			29		
7	15	22	+7	7	1	-6	25	23	−2	47	46	−1	17	28	+11
8	39	49	+10	22	23	+1	42	47	+5	103	119	+16	6	4	−2
9	31	29	−2	10	10	0	40	46	+6	81	85	+4	33	16	−17
11	27			24			32			83			7		
12	15			9			25			49			35		
13	30	23	−7	2	0	−2	20	17	+3	52	40	−12	14	0	−14
15	26	30	+4	13	15	+2	32	37	+5	71	82	+11	18	5	−13

Note: Increased CompACT subscale and total scores represent improved PF. Reduced scores of CSS denote reduced COVID-19-related stress.

## Data Availability

The data presented in this study are available on request from the corresponding author. Raw participant data are presented in Figure 1 and Figure A3, Figure A4, Figure A5 and Figure A6.

## Supplementary Materials

The following supporting information can be downloaded at: https://osf.io/6tr4h/?view_only=9273f2447941494cbedb73c8928bbf70, accessed on 14 March 2022; Study 1 Intervention Protocol; Study 2 Intervention Protocol; Study 2 ACT for IBD Workbook.
